# Editorial: Evolving online modalities: how uses and abuses of text, image and video-based communications impact interpersonal interactions

**DOI:** 10.3389/fpsyg.2024.1476773

**Published:** 2024-09-02

**Authors:** Graham G. Scott, Christopher J. Hand, Gordon P. D. Ingram, Catherine V. Talbot

**Affiliations:** ^1^Department of Psychology, University of the West of Scotland, Paisley, United Kingdom; ^2^School of Education, University of Glasgow, Glasgow, United Kingdom; ^3^School of Science & Technology, RMIT University Vietnam, Ho Chi Minh City, Vietnam; ^4^Department of Psychology, Faculty of Science and Technology, Bournemouth University, Poole, United Kingdom

**Keywords:** online modalities, online social support, social media use, online harm, online wellbeing, text based communication, video communication

Rapid innovations in digital technologies mean that, when interacting online, we are now afforded ways to communicate more quickly and sophisticatedly than ever before. When communicating via electronic text, it is commonplace to use emojis and GIFs to specify meaning and convey emotion. Online communication platforms are also becoming more diverse, meaning that we no longer communicate using only text, but also audio, pictures, video, and a combination of these media. Consequently, online communication is no longer bound by the assumptions of many theories of digital communication which underpin much research in the field. The fact that online communication has become richer and more interactive, and is no longer necessarily asynchronous, means that theories of online communication may need to be expanded or replaced. It is important that we understand how such theories need to evolve with technological advancements, and the implications of how the utilization of new technological affordances impacts users. As ever, online communication devices can benefit or harm both individuals and society depending on how they are used.

Our Research Topic *Evolving online modalities: How uses and abuses of text, image and video-based communications impact interpersonal interactions*, is interested in both how online communication can be positively utilized to nurture relationships and how it may be abused to cause harm. Specifically, the focus is on how different modalities of online communication are employed or exploited in ways not previously investigated. We received seven manuscripts from 27 authors in six countries, covering the sections of Human-Media Interaction, Health Psychology, Media Psychology, and Personality and Social Psychology. The research falls into three areas: (1) online social support; (2) social media use, including how users may manage their social media use to improve aspects of wellbeing; and (3) online harms, with some manuscripts spanning multiple areas.

The first line of research focused on how online social support has evolved to include more modalities. Finn et al. investigated the value of online dance classes as a form of support for young people with anxiety. By applying the Social Cure Framework using both qualitative and quantitative methods, they found that these classes supported participants' holistic wellbeing and allowed them to construct a shared identity, as well as lowering their anxiety, depression, and loneliness, and increasing their self-esteem, self-efficacy and group closeness. Luo et al. used the framework of Ecological System Theory to investigate the relationship between Internet use preferences and loneliness in university students in the context of the COVID-19 pandemic. They found that Internet use preference indirectly influenced loneliness through the mediators of online social support and self-esteem.

The second line of research focused on evolving uses of social media. Mao et al. investigated how users should utilize evolving social media in a beneficial way using Structural Equation Modeling (SEM). Like Luo et al., they were also interested in user loneliness and social media use. Active social media use was related to increased interpersonal satisfaction, and this negatively predicted loneliness and Fear of Missing Out (FoMO). However, active social media use was also associated with increased online-specific state-FoMO, positively predicting trait-FoMO and leading to an increase in loneliness. Elkatmiş used semi-structured interviews to examine the social media usage habits of fourth-grade students in Turkey. Approximately three quarters of participants used social media daily, reporting that they used it for educational, communicative, and entertainment purposes. The most popular platform was YouTube, followed by WhatsApp, TikTok, and Instagram. Qaisar et al. used the Cognition-Affect-Conation (C-A-C) framework to investigate predictors of discontinuous intentions of social media users. Using SEM, they discovered that perceived information overload, perceived social overload, and perceived system feature overload on social networks directly affected depression and anxiety among social networking site users, which directly led to discontinuous intentions. Qin et al. applied Cognitive Load Theory (Sweller, 2023), Social Comparison Theory (Festinger, 1954), and Social Cognitive Theory (Chou et al., 2024) to investigate the antecedents of social media fatigue in a sample of over 650 users. Partial least squares regression analysis revealed that social media self-efficacy negatively impacted social media fatigue; but compulsive social media use, FoMO, and information overload positively impacted social media fatigue.

The final paper focused on online harms. Ramírez-Carrasco et al. investigated factors associated with cyberviolence in dating, focusing on control and humiliation behaviors, as well as intimidation and threats. Analyzing data taken from a sample of over 1,000 individuals, they found that jealousy was related to both victimization from, and perpetration of, cyberviolence, and sexist beliefs were related to perpetration. They replicated previous findings that women were more likely to be victims of cyberdating violence, especially severe violence; with the caveat that their sample was biased toward female participants.

The studies in this Research Topic used a range of qualitative and quantitative methodologies to demonstrate how emerging technologies can have positive impacts on users such as increasing social support, identity, self-esteem, and self-efficacy. However, they can also have negative impacts, leading to increased loneliness, anxiety, social media fatigue, and discontinuous intentions. As technologies evolve, and interactions online become more aligned with interactions offline, more social and cognitive theories are being applied to online interactions (e.g., by Qin et al.). Rather than technologies being viewed as isolated spaces, they are instead being framed as environmental features of interactions. By applying novel theories to online processes (e.g., Social Cure Framework; C-A-C framework; Ecological System Theory) researchers examined how technologies can be used to facilitate beneficial interactions, but how features of these environments may negatively impact, and even deter, users.

Despite the evolution in online modalities toward more “lifelike” forms of communication (on occasions), these articles show that existing concepts in the cyberpsychologist's toolkit—such as online social support, fear of missing out, information overload, and cyberviolence—remain relevant in today's online communication environments. We hope this body of work will provide a platform for researchers to continue investigating communication in the evolving online world, as well as practical guidance for users who navigate constantly developing online platforms.
